# Ultrasonographic characterization of the ulnar collateral ligament of normal thumbs in different age groups

**DOI:** 10.6061/clinics/2018/e162

**Published:** 2018-10-25

**Authors:** Amanda Favaro Cagnolati, Marcel Nader, Marcello Henrique Nogueira-Barbosa, Claudio Henrique Barbieri

**Affiliations:** Departamento de Biomecanica, Medicina e Reabilitacao do Aparelho Locomotor, Faculdade de Medicina de Ribeirao Preto, Universidade de Sao Paulo, Ribeirao Preto, SP, BR

**Keywords:** Thumb, Metacarpophalangeal Joint, Ulnar Collateral Ligament, Ultrasonography

## Abstract

**OBJECTIVES:**

The aim of this study was to perform ultrasonographic characterization of the normal ulnar collateral ligament in different age groups and compare it in men and women and in dominant and nondominant hands.

**METHODS:**

Forty right-handed volunteers in the age groups 20–30, 31–40, 41–50, and 51–60 years without a history of trauma or surgical procedure in the studied joint were evaluated. The studied parameters were ligament length, greatest ligament thickness, ligament longitudinal section area in the longitudinal plane, distance from the aponeurosis of the adductor muscle to the metacarpal head surface and joint opening at rest and under abduction stress.

**RESULTS:**

The results indicated that the mean values of all parameters had minor variations with age, hand dominance, and gender and were slightly higher in men than in women and in the dominant hand than the nondominant hand. However, a statistically significant difference was observed between the joint opening at rest and under stress. In terms of age, there was a small but nonsignificant decrease in the values, likely because of the natural aging process.

**CONCLUSION:**

The low variability in the evaluated parameters indicates that large differences between sides or genders are not to be expected. A greater change is likely to indicate a pathological situation.

## INTRODUCTION

Lesions in the ulnar collateral ligament (UCL) of the thumb are often caused by sports injuries in which forced radial deviation is applied to the abducted thumb. The diagnosis of acute and chronic UCL lesions is usually not difficult based on the patient's history and a clinical examination that reveals a wide, unresisted, sometimes painful passive radial deviation of the metacarpophalangeal joint. However, neither the type nor the extent of the lesion can be confirmed by the clinical findings or by X-ray imaging under stress. An accurate diagnosis, including lesion type and extent, can be made using ultrasonographic examination, which has demonstrated a compatibility with intraoperative findings that ranges between 87% and 92% 1. Arthrography and magnetic resonance imaging are also used for the same purpose, but ultrasonography has some advantages over other tests, such as low cost, availability, dynamicity and speed. Furthermore, ultrasonography is painless and free of ionizing radiation 2,3. Its disadvantage is that its accuracy depends on examiner ability, equipment quality, correct positioning of the transducer, and the interface used 4.

Ultrasonographic examinations of the UCL are conducted almost exclusively in pathological situations, when gross changes to the ligament anatomy have already occurred. In addition, the normal UCL has not been characterized, and its differences among genders, age groups, and hand dominance have not been evaluated. Therefore, this study aimed to evaluate the normal UCL and provide ultrasonographic parameters that will allow comparisons between normal and injured UCLs.

## MATERIALS AND METHODS

From May to December 2009, ultrasonography was performed on the thumbs of 40 healthy volunteers (21 women and 19 men) in the age groups 20–30 years (group 1), 31–40 years (group 2), 41–50 years (group 3), and 51–60 years (group 4), with approximately 10 subjects in each group. Although all the subjects were right-handed, both hands were examined. The inclusion criteria were age between 20 and 60 years, absence of a history of trauma in the evaluated joint, and not having undergone any surgical procedure on the evaluated thumbs.

**Figure 1:**
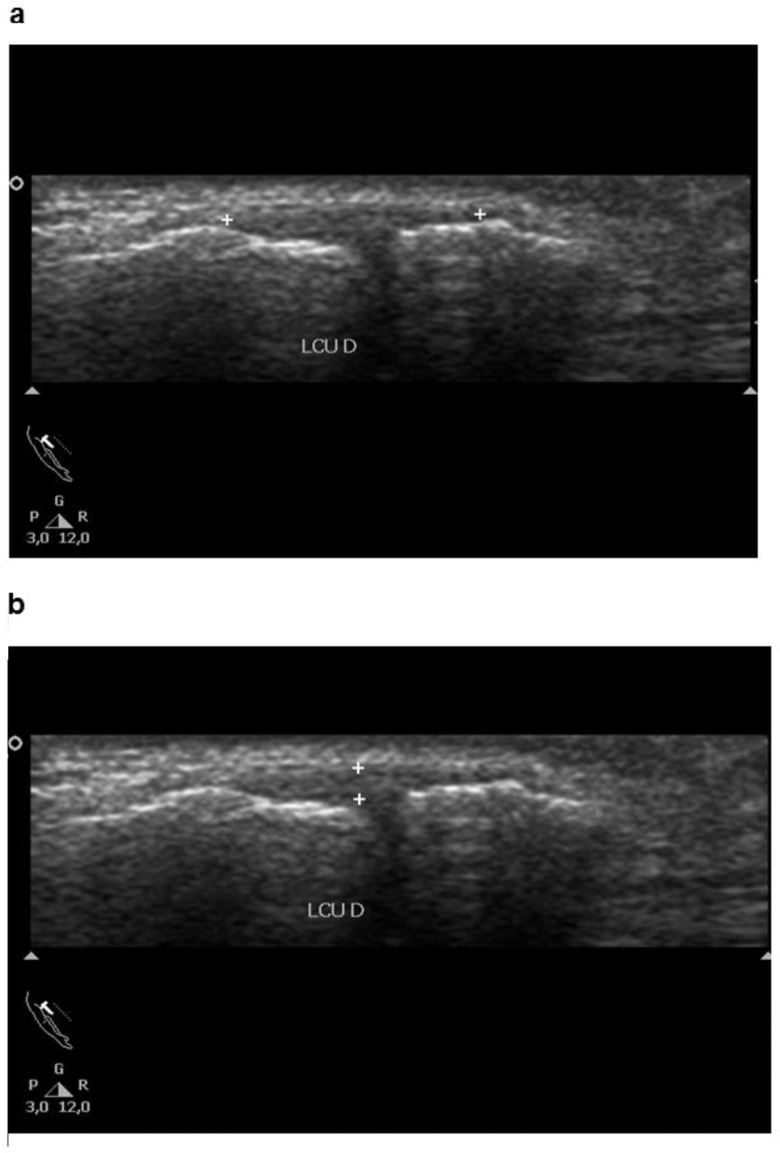
Ultrasonographic images showing the length (a) and thickness (b) of the collateral ulnar ligament of the thumb.

**Figure 2:**
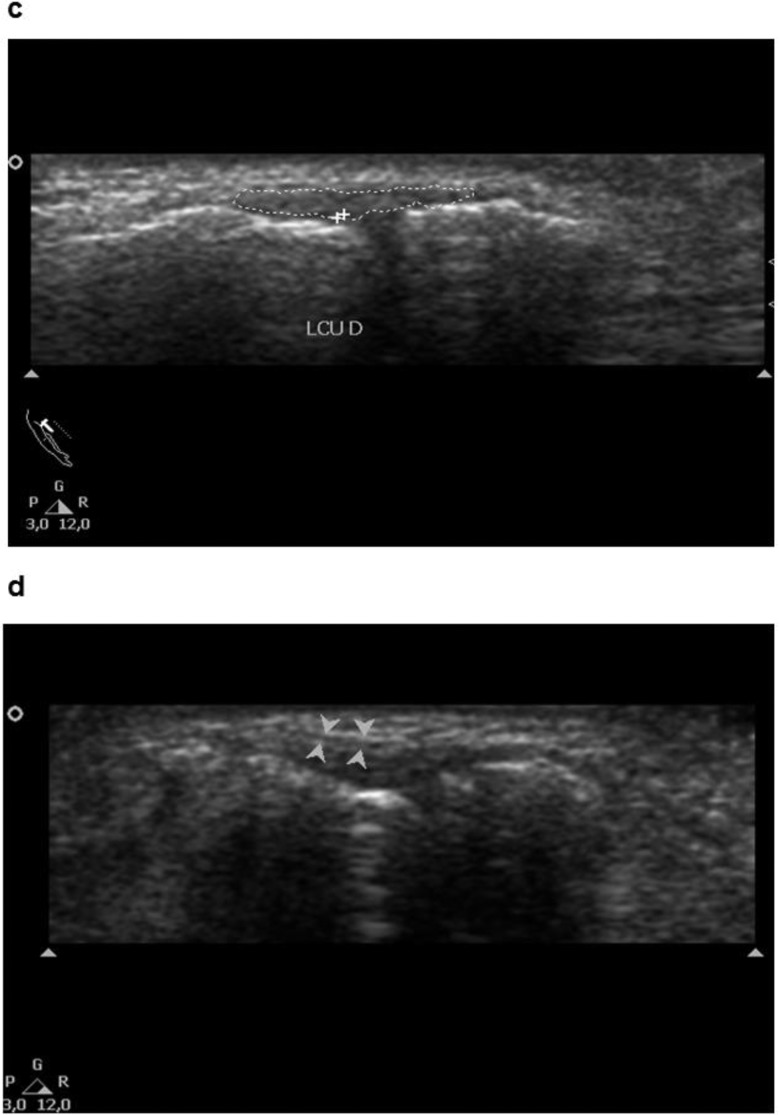
Ultrasonographic images showing the perimeter area of the ligament (c) and aponeurosis of the adductor pollicis muscle (d).

**Figure 3:**
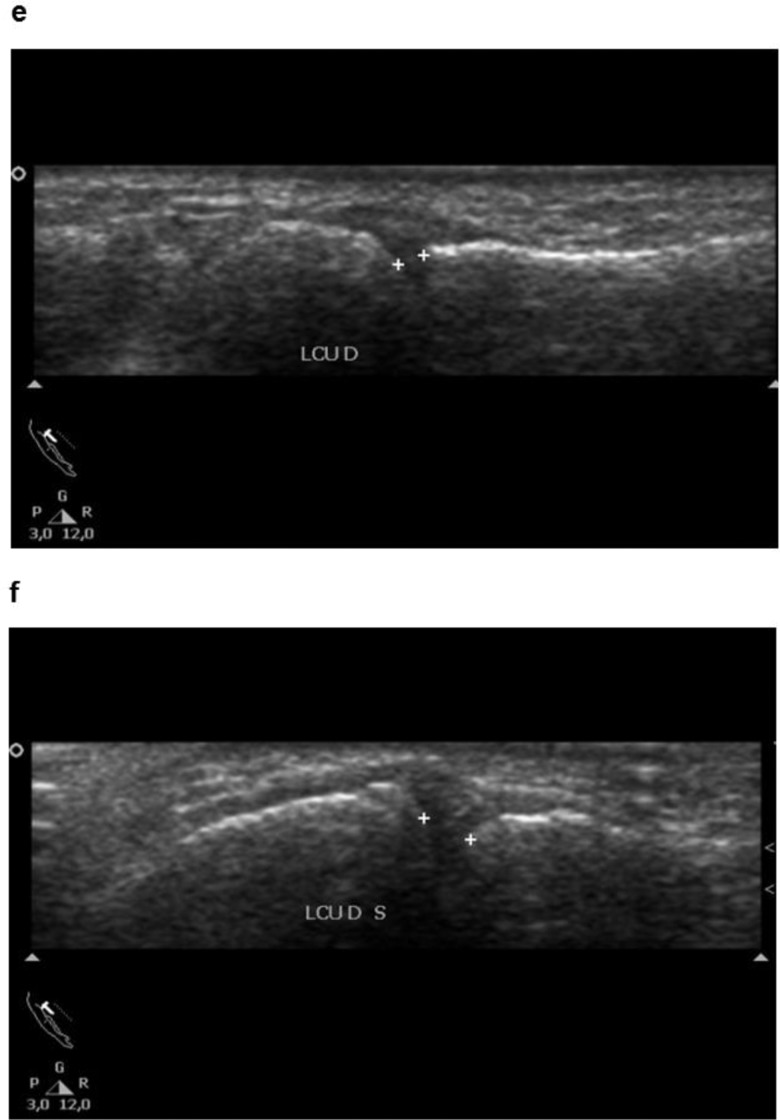
Ultrasonographic images showing the articulation opening of the metacarpophalangeal joint in the resting position (e) and under stress (f).

All examinations were conducted by the same radiologist with previous training and a fellowship in musculoskeletal radiology (MN), and all patients were followed by a hand surgeon (AFC). The real-time ultrasonography equipment used was coupled with a 15-MHz transducer (HD 11 Ultrasound System, Philips Medical Systems, Ltd., Bothell, Washington, USA). The examination was performed on the right hand and then on the left hand, and abundant surface coupling gel was used. Images from two longitudinal planes centered on the metacarpophalangeal joint were obtained, including one oblique plane in the direction of the axis of the main fibers of the UCL and another plane parallel to the main axis of the bones. In the oblique images, the morphological variables measured were ligament length, ligament thickness, ligament longitudinal sectional area, and distance from the aponeurosis of the adductor tendon to the surface of the head of the metacarpal bone. In the images parallel to the long axis of bones, the variable measured was the opening of the joint cleft at rest and during a stress maneuver, with full forced abduction of the thumb performed by the surgeon (Figures 1-3).

The data were submitted to statistical analysis using a mixed-effects linear model adjusted by the Proc Mixed procedure of SAS v.9 software at the 5% level of significance (*p*≤0.05). An analysis of residues was conducted to check assumptions, and the logarithmic transformation was found to be adequate to satisfy this demand in some cases. A raw comparison between average values was also undertaken for each situation 5.

### Ethics

The study was approved by the Ethics in Human Research Committee, and all participants gave written informed consent.

## RESULTS

The general data for the analyzed parameters are shown in Tables 1, 2 and 3, and the results for the right and left hands are presented separately.

### Right hand (dominant)

The mean lengths of the UCL were 9.1, 10.2, 9, and 10.6 mm in age groups 1, 2, 3, and 4, respectively, and the differences were not significant for all comparisons (1 *vs*. 2, 1 *vs*. 3, 1 *vs*. 4, 2 *v*s. 3, 2 *vs*. 4, and 3 *vs*. 4; *p*=0.75–0.98). In all age groups, the mean length of the UCL was 9.7 mm in women and 10.5 mm in men, and the gender differences were not significant (*p*=0.59).

The mean thickness of the UCL was 1.6 mm for age group 1 and 1.7 mm for the other age groups, and the differences were not significant for all comparisons (*p*=0.28–0.95). In all age groups, the mean thickness was 1.6 mm in women and 1.7 mm in men, and the gender differences were not significant (*p*=0.61).

The mean longitudinal sectional area of the UCL was 1 mm^2^ in age groups 1, 2, and 3, respectively, and 1.1 mm^2^ in age group 4, and the differences were not significant for all comparisons (*p*=0.34–0.85). The mean perimeter area was 1 mm^2^ in women and men, without significant difference between the genders (*p*=0.47).

The mean distance from the aponeurosis of the adductor muscle of the thumb to the head of the metacarpal bone was 1.6 mm in age groups 1, 3, and 4 and 1.7 mm in age group 2, without significant differences for all comparisons (*p*=0.32–0.94). This distance was 1.7 mm in women and 1.6 mm in men, without significant differences between the genders (*p*=0.48).

The mean opening of the metacarpophalangeal joint at rest was 1.7 mm in age group 1, 1.6 mm in age group 2, and 1.5 mm in age groups 3 and 4, and the differences were significant only for the comparison between groups 1 and 4 (*p*=0.02). The mean opening was 1.5 mm in women and 1.7 mm in men, and the difference between the genders was significant (*p*=0.01).

Under ulnar stress, the mean opening of the metacarpophalangeal joint increased to 2.5, 2.4, 2.2, and 2.1 mm in age groups 1, 2, 3, and 4, respectively, but the differences among the groups were not significant for all comparisons (*p*=0.06–0.74). The opening was 2.1 mm in women and 2.5 mm in men, with significant differences between the genders (*p*<0.01).

### Left hand

The mean UCL lengths were 9.1 mm, 10.2 mm, 9 mm, and 9.3 mm for age groups 1, 2, 3, and 4, respectively; however, no significant differences among the groups were observed (*p*=0.24–0.99). The average length was 8.4 mm in women and 10.5 mm in men, with a significant difference between genders (*p*=0.01).

The mean UCL thickness was 1.7 mm for age groups 1, 3, and 4 and 1.6 mm for age group 2, without significant differences between the groups (*p*=0.35–0.99). The mean UCL thickness was 1.6 mm in women and 1.8 mm in men, with no significant difference between the genders (*p*=0.13).

The mean UCL perimeter area was 1 mm^2^ in age groups 1, 2, and 3 and 1.1 mm^2^ in age group 4. There was no significant difference among the groups (*p*=0.34–0.85). The mean UCL perimeter area observed in women was 1 mm^2^, and that observed in men was 1.1 mm^2^; no significant difference between genders was found (*p*=0.13).

The mean distance between the aponeurosis of the adductor pollicis muscle and the metacarpal head was 1.7 mm in age groups 1 and 3 and 1.6 mm in age groups 2 and 4. The differences among the age groups were not significant (*p*=0.45–0.96). The mean distance was 1.5 mm in women and 1.8 mm in men, and the difference between genders was significant (*p*=0.03).

The mean opening of the metacarpophalangeal joint in the resting position was 1.7 mm for age groups 1 and 4, 1.8 mm for age group 2, and 1.6 for age group 3. There was a significant difference between groups 2 and 3 (*p*=0.01). The mean joint opening observed in women was 1.6 mm, and that observed in men was 1.8 mm, with a significant difference between genders (*p*=0.01).

The mean opening of the metacarpophalangeal joint under ulnar stress increased to 2.4 mm for age group 1, 2.5 mm for age group 2, 2.3 mm for age group 3, and 2.2 mm for age group 4. No significant differences among age groups were observed (*p*=0.17–0.84). The mean articulation opening observed in women was 2.2 mm, and that observed in men was 2.5 mm, with a significant difference between genders (*p*=0.03).

Overall, the differences between the left and right hand parameters were only significant in relation to articulation opening in the resting position (*p*=0.01; Table 3).

## DISCUSSION

Ultrasonography is highly accurate in identifying ligament ruptures, and it can differentiate among disruptions, in situ ruptures and Stener lesions. This assessment method is fast, inexpensive, dynamic, easily available, and completely painless. However, its accuracy depends on the skill and experience of the examiner. Although some authors state a preference for arthrography or magnetic resonance imaging 6, the sensitivity of ultrasonography is 88%, and its specificity is 91%. Exam failure may occur as a result of certain factors, such as the condition of the patient's skin, equipment quality, and operation mode. For example, excessive transducer pressure interferes with the quality of the images, which can lead to false diagnosis. In addition, the test's accuracy decreases after the first week because the healing process affects the images after this period 4.

Previous UCL imaging studies were performed in pathological situations in which great anatomical changes of the ligament had already occurred; this allowed characterization by ultrasonography, especially after comparisons with surgical findings or other diagnostic methods, such as X-rays under stress conditions, arthrography, and magnetic resonance imaging. However, to the best of our knowledge, no study has focused on the ultrasonographic characterization of the normal UCL to provide comparative data, particularly in cases in which the lesion is not very extensive. This was the motivation for the present study, which specifically addressed the proper proportions of the UCL.

**Figure 4:**
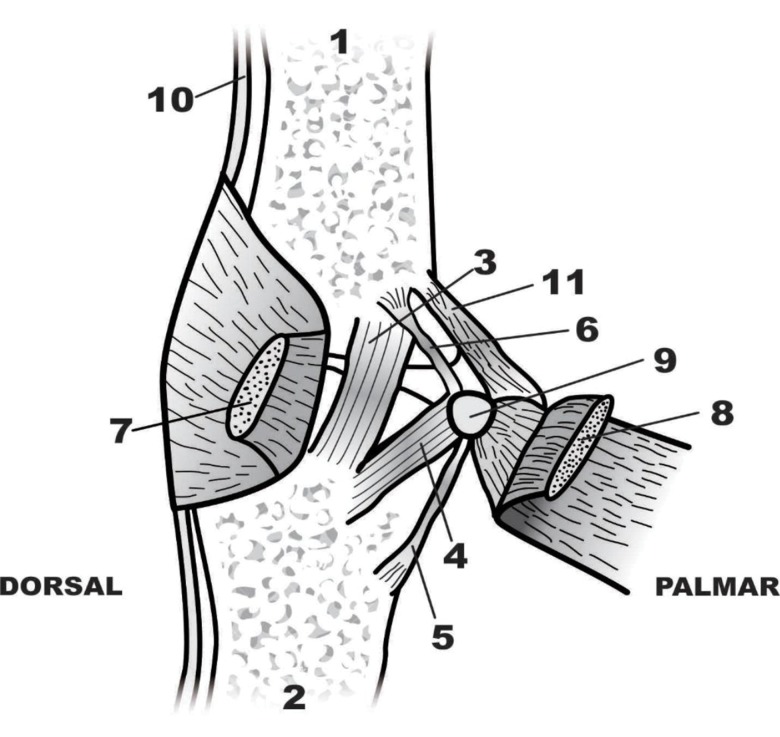
Anatomy of the metacarpophalangeal joint from the ulnar side. 1: proximal phalanx, 2: first metacarpal, 3: UCL, 4: AUCL, 5: palmar proximal ligament, 6: volar, 7: aponeurosis of the adductor, 8: adductor pollicis muscle, 9: sesamoid ulnar bone, 10: extensor tendon, and 11: abductor tendon of the thumb.

**Figure 5:**
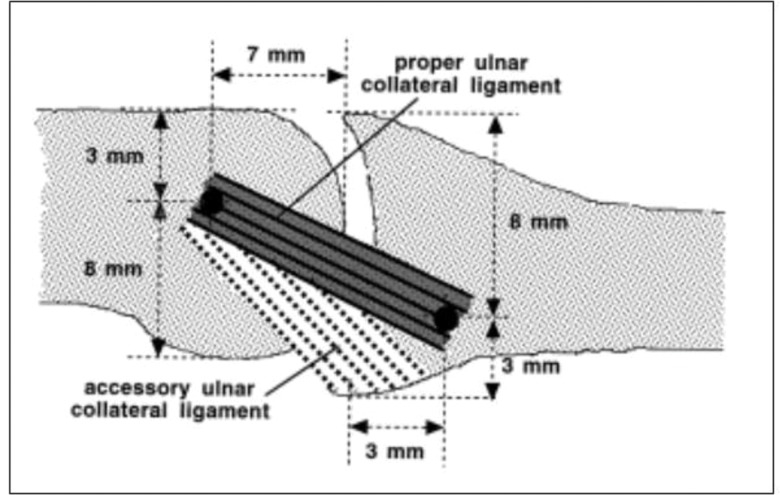
Origin and insertion of the UCL and the metacarpophalangeal joint of the thumb.

A normal ligament is identified by ultrasonography as a narrow and convex hyperechoic fibrous band that extends from the metacarpal head to the base of the proximal phalanx, similar to images from anatomical studies of cadavers. Such images defined the origin, insertion, and mean length and width of the UCL and were used as the basis for further studies 1,7. The metacarpophalangeal joint of the thumb is a ginglymus-type diarthrosis that is statically stabilized by strong ligaments 1,8. The UCL of the thumb originates in the head of the first metacarpal bone, passes from a proximal dorsal position to a distal and palmar position, and inserts into the ulnar tubercle of the base of the proximal phalanx. It is 12–14 mm long and 4–8 mm wide 2 and includes the UCL and an accessory UCL (AUCL). The AUCL originates in the same region as the UCL and inserts into the volar capsule and aponeurosis of the adductor muscle, which broadly covers the UCL–AUCL complex (Figure 4) 1. Studies of cadavers established the limits of the origin and insertion of the UCL, and these values have been used as guides. The center of the origin of the UCL in the head of the metacarpal bone is located approximately 7 mm from the distal margin of the joint, 3 mm from the dorsal margin, and 8 mm from the palmar border. The center of the insertion in the proximal phalanx is located 3 mm from the proximal margin of the joint, 3 mm from the palmar margin, and 8 mm from the dorsal margin (Figure 5) 7. The function of the UCL–AUCL complex varies with the position of the metacarpophalangeal joint; stability is provided by the AUCL during extension and by the UCL during flexion 7. Both offer dorsal support and prevent palmar subluxation of the proximal phalanx 2. The loss of stability of the ulnar side of the joint compromises the lateral pinch, alters the biomechanics of the metacarpophalangeal joint, and predisposes the individual to premature degenerative arthritis.

The scope of this study was to characterize the normal UCL in men and women of different age groups using ultrasonography and not in the individuals in whom lesions are more likely to occur. Although this may represent a drawback, statistical planning was not performed because there was no similar paper on the literature that could be used as a model, in accordance with the statistician's preference; the *n* was thus fixed at 10 volunteers per group following her suggestion. The fact that all the patients were right-handed was a mere coincidence and not an criterion. The results showed that in general, the variation in the analyzed parameters was relatively small, and almost no significant differences among age groups or between genders were observed. Differences for the right hand (dominant) only were significant (*p*=0.01) for the articulation opening in the resting position between age groups 1 and 4; the opening was greater in younger individuals. This may be due to the greater thickness of the articular cartilage, which decreases with age and thus decreases the articulation opening in older individuals. For the left hand, there were also significant differences (*p*=0.01) for the articulation opening, but only between age groups 2 and 3. The reason for this may also be related to differences in the thickness of the articular cartilage.

Regarding gender, differences were significant for the opening in the resting position for the right and left hands (*p*=0.01 for both). The same pattern was observed during opening under stress conditions (*p*<0.01 for the right; *p*=0.03 for the left). In addition to these parameters, there were significant differences between genders for the UCL length (*p*=0.01) and for the distance of the aponeurosis of the adductor pollicis muscle (*p*=0.03) but only in the left hand. In general, for both the right and left hands, the ulnar opening under stress conditions tended to considerably decrease; however, no direct relationship was observed with the UCL length.

Despite the relatively small population sample, we could conclude that the low variability in the evaluated parameters indicates that large differences between sides or genders are not to be expected. A larger change is likely to indicate a pathological situation.

## AUTHOR CONTRIBUTIONS

Cagnolati AF selected volunteers, conducted the examinations and interpreted the preliminary data. Nader M performed all ultrasonographic examinations. Nogueira-Barbosa MH selected ultrasound parameters and reviewed the collected images. Barbieri CH designed the study, reviewed the collected data and prepared the English version of the manuscript.

## Figures and Tables

**Table 1:** Comparison of the parameters of the right hand between genders (M-F).

Parameters	Dif	I. 95%CI	S. 95%CI	*p*-value
Length*	-0.08	-0.36	0.21	0.59
Thickness*	-0.03	-0.14	0.08	0.61
Area*	-0.06	-0.21	0.10	0.47
Distance from aponeurosis*	0.04	-0.08	0.16	0.48
Joint opening*	-0.12	-0.21	-0.03	0.01
Joint opening with stress*	-0.18	-0.29	-0.08	<0.01

*logarithm transformation was applied.

**Table 2:** Comparison of the parameters of the left hand between genders (M-F).

Parameters	Dif	I. 95%CI	S. 95%CI	*p*-value
Length*	-0.41	-0.69	-0.13	0.01
Thickness*	-0.08	-0.19	0.03	0.13
Area*	-0.12	-0.28	0.04	0.13
Distance from aponeurosis*	0.13	-0.25	-0.01	0.03
Joint opening*	-0.12	-0.21	-0.04	0.01
Joint opening with stress*	-0.12	-0.22	-0.02	0.03

*logarithm transformation was applied.

**Table 3:** Comparison of the parameters between the right and left hands.

Parameters	Dif	I. 95%CI	S. 95%CI	*p*-value
Length*	0.18	-0.02	0.38	0.08
Thickness*	-0.01	-0.08	0.06	0.77
Area*	-0.02	-0.10	0.05	0.53
Distance from aponeurosis*	-0.02	-0.09	0.06	0.70
Joint opening*	-0.07	-0.12	-0.02	0.01
Joint opening with stress*	-0.04	-0.10	0.02	0.18

*logarithm transformation was applied.
